# Can Echocardiography Be Used for Screening of Rheumatic Heart Disease?

**DOI:** 10.4103/0970-0218.45386

**Published:** 2009-01

**Authors:** Harshal Tukaram Pandve, JS Bhawalkar, Amitav Banerjee, Pankaja Bhuyar

**Affiliations:** Department of Community Medicine, Dr. DY Patil Medical College, Pimpri, Pune - 411 018, Maharashtra, India. E-mail: dr_harshalpandve@yahoo.co.in

Sir,

In poor and developing nations, rheumatic heart disease remains a major cause of morbidity and premature death and imposes a substantial burden on health care systems with limited budgets.(1) Secondary prevention is one of the effective tools in rheumatic heart disease. Secondary prevention relies on accurate case detection for the appropriate use of prophylactic antibiotics and regular medical surveillance.

Traditionally, cases of rheumatic heart disease are identified by clinical examination only, while echocardiography is used for confirmation of the same. Almost all population-based epidemiologic surveys have relied on this strategy. Such surveys show current prevalence rates of rheumatic heart disease of approximately 1 to 5 cases per 1000 among school-age children in developing countries.(2)

Echocardiography is known to be more sensitive than auscultation for the detection of pathologic valve disease. Meira *et al*, reported that in 34% of their patients with carditis, clinical examination after the acute phase showed normal findings, although progression to chronic sub clinical valvular disease was confirmed by echocardiography in 82% of such cases.(3) According to Marijon *et al*. systematic screening with echocardiography, as compared with clinical screening, reveals a much higher prevalence of rheumatic heart disease.(4)

World Health Organization (WHO),(5) states that, echocardiographically diagnosed, clinically silent rheumatic valve involvement should be managed as rheumatic heart disease until proved otherwise. WHO also states that, it is important to recognize that technical expertise with color flow Doppler echocardiography is necessary to make an accurate diagnosis of sub clinical carditis and to avoid over diagnoses. In developing countries, where the majority of rheumatic fever (RF) cases occur, such expertise and facilities are available in only a limited number of centres. As a result, the impact of erroneous diagnoses of rheumatic carditis based on sub clinical echocardiographic findings should not be underestimated, nor should the potentially adverse consequences to patients and health systems in such settings. High sensitivity of Doppler echocardiography is also questionable in using it as screening test. Prevalence of carditis in RF patients is significantly higher when detected by echocardiography than that reported clinically, and the utility of a test that diagnoses a disease characteristic (such as carditis in RF) in almost every patient with RF is questionable.(5) Clinically suspected cases of rheumatic heart disease are confirmed by echocardiography, but how to confirm the cases detected by echocardiography?

From statistical viewpoint, Bayes theorem states that the probability of disease following the interpretation of a diagnostic investigation (the post-test probability) is determined by two factors. Firstly, the probability of disease prior to carrying out the investigation (the pre-test probability) based on the information already available and secondly the characteristics of the test. Any decision for screening strategy should ideally entail the calculation of prior probability (i.e., prevalence) of rheumatic heart disease in a given area along with diagnostic accuracy (i.e., sensitivity and specificity) of screening criteria (clinical) or method (echocardiography) so as to have correct posterior probability of rheumatic heart disease.

These are some of the issues which have to be considered before using echocardiography as screening tool for detection of rheumatic heart disease in large populations.
